# Supplementary material for: The impact of socio-economic institutional change on agricultural carbon dioxide emission reduction in China
Source: PLoS One. 2021 May 19;16(5):e0251816. doi: 10.1371/journal.pone.0251816 (PMC8133459; doi:10.1371/journal.pone.0251816)
Supplement: Attachment — Submitted filename: PONE-D-20-35559 (12.16.20).pdf [file pone.0251816.s002.pdf]

Manuscript #: PONE-D-20-35559
Title: The Impact of Socio-economic Institutional Change on Agricultural Carbon
Authors: Long & Tang

The manuscript by Long and Tang aims to use the "EKC model to analyze economic growth and agricultural carbon dioxide emissions in the perspective of ecological civilization construction". Their experimental results show that the relationships between economic growth and the total intensity of agricultural carbon emissions, and between economic growth and the intensity of carbon emissions caused by five types of carbon sources: fertilizer, pesticide, agricultural film, agricultural diesel oil and tillage are integrated. In addition, economic growth was identified as the main driving factor of agricultural carbon dioxide emissions. I found this manuscript very difficult to follow. At the outstart I was confused by the term "ecological civilization construction" and still I am not clear on what this means. Furthermore, I found that the findings of the work were not surprising and couldn't help but think throughout reading the manuscript, that we already know much of what the authors are claiming to reveal. Which brings me to what is significant in this research and manuscript. I also found that when I referred to the literature that was cited, in many cases I could not fathom how the citation was related to the point being made (e.g., #7). Many of the references seem to be either obscure or perhaps incorrect. Overall, then, I do not recommend that this manuscript be published in PlosOne and at a minimum it requires a major revision. A few small comments:

1. What is an agricultural film (p. 10) and how does its use produce CO_2 emissions?
2. Why not present the data in Table 2 graphically – I think it would be much more effective and communicate your point better.
3. Page 15 – is it necessary to spell out all the logs of CO_2 emissions and real per capita GDP?
4. Table 8 – is there a more compelling way to present these date and to identify trends – is there a trend?
